# Supplementary material for: Content-rich biological network constructed by mining PubMed abstracts
Source: BMC Bioinformatics. 2004 Oct 8;5:147. doi: 10.1186/1471-2105-5-147 (PMC528731; doi:10.1186/1471-2105-5-147)
Supplement: Additional File 5 — The original Chilibot query results of the term "long-term potentiation (LTP)" and 22 other terms, limiting the latest references analyzed to the years 1990, 1995, 2000, and 2004. [file 1471-2105-5-147-S5.bz2 › chilibotAdditionalFile5/ltp2004/html/ACTIN.html]

 


**ACTIN** (Input: ACTIN ) 

---


**Google Searches:** Entire Web  | EDU domain only  | PDF files only

.

**External Links:** OMIM |
LocusLink
| Swissprot
| GeneCards

  
**Maps of ACTIN**

Simple
Complete
graph in
radiant
tree
square
layout.

**New Hypothesis !**

**Synonyms** 

- actin   [PubMed]

**Synopsis**

- The data obtained support our recent suggestion on the role of filamentous **actin** in induction and long term maintenance of enhanced electrotonic conductivity at mixed synapses.  Tsitologiia, 1999 
  [23]

- Our results strongly suggest that syndapins link endocytosis and **actin** dynamics.  J Cell Biol, 2000 
  [23]

- These results suggest that postsynaptic **actin** filaments are involved in a dynamic process required to maintain AMPAR mediated transmission and to enhance it during LTP.  J Neurosci, 1999 
  [23]

- The results are consistent with a model in which dynamic **actin** filaments play an essential role in the molecular mechanisms underlying the early maintenance phase of LTP, such as growth of new synaptic connections or conversion of silent synapses.  Proc Natl Acad Sci U S A, 2000 
  [20]

- These results indicate that the accumulation of Homer1a, either exogenously expressed or endogenously induced, caused redistribution and dispersion of postsynaptic clusters of Homer1c and F **actin**, suggesting an important role of Homer1a in synaptic remodeling.  Neurosci Lett, 2004 
  [20]

- As synaptopodin is an **actin** associated protein present in spine neck and implicated in the modulation of cell morphology, our results suggest that synaptopodin, by regulating the dynamics of the **actin** cytoskeleton, contributes to the morphological change in spine shape considered to be important for the maintenance of synaptic plasticity.  J Neurochem, 2001 
  [20]

- These observations suggest that mechanisms regulating the spine **actin** cytoskeleton contribute to the persistence of LTP.  Neuron, 2003 
  [19]

- Using LIMK 1 knockout mice, we demonstrate a significant role for LIMK 1 in vivo in regulating cofilin and the **actin** cytoskeleton.  Neuron, 2002 
  [19]

- DLS contribution to electrotonic transmission is suggested, direct or mediated by **actin** interaction with nearby localized GJ connexons.  Morfologiia, 1998 
  [19]

- After training, pp60c src from hippocampus showed enhanced interactions with synaptic proteins such as synapsin I, synaptophysin, and the type 2 N methyl d aspartate receptor, as well as the cytoskeletal protein **actin**.  Proc Natl Acad Sci U S A, 2000 
  [16]

- Synaptic vesicles in the reserve pool are thought to be reversibly tethered to the **actin** based cytoskeleton by the synapsins, a family of synaptic vesicle associated phosphoproteins that have been shown to play a role in the formation, maintenance, and regulation of the reserve pool of synaptic vesicles and to operate during the post docking step of the release process.  J Neurosci, 2001 
  [16]

- Cytochalasin D, which disrupts the **actin** cytoskeleton, blocked ACPD elicited chloride currents and ACPD induced potentiation of NMDAR currents, consistent with a role for cytoskeletal protein in the signaling pathway.  Neuropharmacology, 2001 
  [16]

- We suggest how recently discovered binding interactions could provide a structural readout mechanism the autophosphorylated state of CaMKII binds tightly to the NMDAR and forms, through CaMKII actinin **actin** 4.1 SAP97 linkages, additional sites for anchoring AMPARs at synapses.  Neuron, 2001 
  [16]

- The SH3 mediated interaction of synapsin I with amphiphysins affected the ability of synapsin I to interact with **actin** and synaptic vesicles, and pools of synapsin I and amphiphysin I were shown to associate in isolated nerve terminals.  J Biol Chem, 2000 
  [15]

- While all the anti synapsin I mAbs were unable to affect the interactions of synapsin I both with calcium calmodulin dependent protein kinase II and with **actin** monomers and filaments, mAbs 19.8 and 19.51 were found to inhibit the binding of Grb2 SH3 domains to the proline rich C terminal region of synapsin I.  Brain Res Mol Brain Res, 1997 
  [15]
